# Post-transcriptional control of miRNA biogenesis

**DOI:** 10.1261/rna.068692.118

**Published:** 2019-01

**Authors:** Gracjan Michlewski, Javier F. Cáceres

**Affiliations:** 1 Division of Infection and Pathway Medicine, University of Edinburgh, Edinburgh EH16 4SB, United Kingdom; 2 Zhejiang University-University of Edinburgh Institute, Zhejiang University, Zhejiang 314400, P.R. China; 3 MRC Human Genetics Unit, Institute of Genetics and Molecular Medicine, University of Edinburgh, Western General Hospital, Edinburgh EH4 2XU, United Kingdom

**Keywords:** microRNAs (miRNAs), terminal loop, Microprocessor, DGCR8, DROSHA, DICER, RNA-binding proteins (RBPs)

## Abstract

MicroRNAs (miRNAs) are important regulators of gene expression that bind complementary target mRNAs and repress their expression. Precursor miRNA molecules undergo nuclear and cytoplasmic processing events, carried out by the endoribonucleases DROSHA and DICER, respectively, to produce mature miRNAs that are loaded onto the RISC (RNA-induced silencing complex) to exert their biological function. Regulation of mature miRNA levels is critical in development, differentiation, and disease, as demonstrated by multiple levels of control during their biogenesis cascade. Here, we will focus on post-transcriptional mechanisms and will discuss the impact of *cis*-acting sequences in precursor miRNAs, as well as *trans*-acting factors that bind to these precursors and influence their processing. In particular, we will highlight the role of general RNA-binding proteins (RBPs) as factors that control the processing of specific miRNAs, revealing a complex layer of regulation in miRNA production and function.

## INTRODUCTION

MicroRNAs (miRNAs) are small noncoding RNAs that negatively regulate the expression of a large proportion of cellular mRNAs. They have unique, diverse expression patterns (Landgraf et al. 2007) and affect many cellular processes and developmental pathways (Ebert and Sharp 2012; Bartel 2018). Most miRNA genes are transcribed by RNA polymerase II (Pol II), with the long primary transcript, termed pri-miRNA, harboring a hairpin structure, which comprises the miRNA sequence. Whereas many of these genes are transcribed as intronic clusters within protein-coding pre-mRNAs, others can be transcribed as independent gene units, or be encoded within long noncoding RNAs (lncRNAs) (Rodriguez et al. 2004; Kim and Kim 2007).

The biogenesis of miRNAs is carried out by two RNase III enzymes, DROSHA and DICER, which catalyze two subsequent processing events, in the nucleus and in the cytoplasm, respectively (Hutvágner et al. 2001; Ketting et al. 2001; Lee et al. 2003). The nuclear event is catalyzed by the Microprocessor complex, which comprises the RNase III type enzyme DROSHA, the double-stranded RNA-binding protein (RBP) DGCR8 (DiGeorge syndrome critical region 8 gene) and associated proteins (Denli et al. 2004; Gregory et al. 2004; Han et al. 2004; Landthaler et al. 2004). This nuclear processing event results in the production of ∼70 nucleotide (nt) stem–loop precursor miRNAs, termed pre-miRNAs (Han et al. 2004; Zeng et al. 2005), which are subsequently exported to the cytoplasm using the export receptor, Exportin-5 (Yi et al. 2003; Bohnsack et al. 2004; Lund et al. 2004). Once in the cytoplasm, pre-miRNAs undergo a final processing event, by another RNase type III enzyme, DICER, to give rise to miRNA duplexes (Hutvágner et al. 2001; Ketting et al. 2001). These are then incorporated into the RISC (RNA-induced silencing complex) together with an Argonaute (AGO) protein, where one strand is selected to become the mature miRNA (Kobayashi and Tomari 2016). In addition, there are also noncanonical miRNA biogenesis pathways that lead to the production of functional miRNAs. These include mirtrons that are generated via pre-mRNA splicing and miRNAs generated from small nucleolar RNA (snoRNAs) precursors (for review, see Ha and Kim 2014). Regulation of gene expression by miRNAs is also prevalent in plants; however, several aspects of their biogenesis and function differ (for comprehensive reviews, see Axtell et al. 2011; Naqvi et al. 2012; Bologna et al. 2013). Several excellent recent reviews have focused on the function of animal miRNAs (Bracken et al. 2016; Bartel 2018; Gebert and MacRae 2018). Here, we will focus on post-transcriptional mechanisms that regulate miRNA production in animals, with a particular focus on the role of RBPs in the post-transcriptional regulation of their biogenesis.

### Nuclear step of miRNA processing: the Microprocessor

**FIGURE 1.**
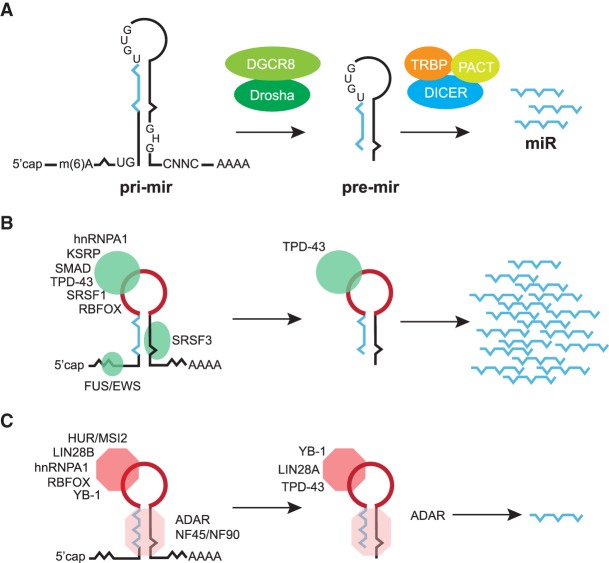
(*A*) The canonical pathway of miRNA biogenesis, including the Microprocessor-mediated (DROSHA/DGCR8) step in the nucleus followed by DICER processing in the cytoplasm. Structural and sequence features important for miRNA processing are highlighted in both the pri-mir and pre-miR molecules. (*B*) Positive regulators of miRNA biogenesis bind to the terminal loop (TL) or other elements within miRNA precursors (pri-mir and pre-mir) and stimulate Drosha and/or Dicer processing, leading to increased levels of mature miRNAs (miR). The TL, also known as apical loop, is depicted in red. (*C*) Negative regulators of miRNA biogenesis bind to TL or other elements within miRNA precursors (pri-mir and pre-mir) and abrogate Drosha and/or Dicer processing, leading to decreased levels of mature miRNAs (miRs). The TL, also known as apical loop, is depicted in red.

The nuclear phase of miRNA processing occurs cotranscriptionally acting on both independently transcribed and intron-encoded miRNA (Morlando et al. 2008). This cotranscriptional processing can be facilitated by HP1BP3, a histone H1-like chromatin protein, which interacts with both the Microprocessor and endogenous pri-miRNAs to promote cotranscriptional miRNA biogenesis in human cells (Liu et al. 2016). MiRNA precursors form RNA hairpins that need to be recognized by the Microprocessor. To distinguish primary miRNAs (pri-miRNAs) from other hairpin-containing transcripts, additional identifiers are required. These determinants comprise a ∼35 bp stem harboring a mismatched GHG motif and also include three primary-sequence elements, a basal UG motif, an apical UGUG motif and a CNNC motif, which binds the SR protein, SRSF3, and is found downstream from approximately 60% of all pri-miRNA hairpins (Fig. 1A; Auyeung et al. 2013; Fang and Bartel 2015). Another proposed identifier of what constitutes a bona fide pri-miRNA stem–loop is the presence of an *N*6-methyladenosine (m^6^A) mark in the vicinity of the pri-miRNA stem–loop. This mark is bound by a reader, the hnRNP protein, A2/B1, which interacts with DGCR8 and stimulates miRNA processing (Alarcón et al. 2015a, 2015b; Knuckles et al. 2017).

The precise mechanism by which the Microprocessor recognizes pri-miRNAs and catalyzes their processing is beginning to be fully understood. In brief, the Microprocessor is a heterotrimeric complex, comprising one DROSHA and two DGCR8 molecules. The DGCR8 dimer interacts with the stem and apical elements of the pri-miRNAs through its double-stranded RNA-binding domains (dsRNA) and RNA-binding heme domain, respectively, leading to accurate and efficient processing. In contrast, DROSHA serves as a ruler to measure an 11 base pair (bp) distance from the basal single-stranded RNA-double stranded RNA (ssRNA-dsRNA) junction and cleaves the stem–loop of primary miRNAs (Nguyen et al. 2015). Crucially, the orientation of this complex on the substrate is maintained by DROSHA and DGCR8 recognizing the basal UG and apical UGUG motifs, respectively (Nguyen et al. 2015; Kwon et al. 2016). It has been recently shown that SRSF3, a member of the SR protein family of splicing factors promotes miRNA processing by recruiting DROSHA to the basal junction in a CNNC-dependent manner (Kim et al. 2018). The activity of the Microprocessor can be enhanced by the binding of heme, a ferric ion-containing porphyrin, which promotes the interaction between the DGCR8 dimer and the apical UGUG motif, promoting Microprocessor activity (Quick-Cleveland et al. 2014; Weitz et al. 2014; Partin et al. 2017; Nguyen et al. 2018).

### Noncanonical functions of the Microprocessor

Besides its established role in miRNA biogenesis, noncanonical functions for the Microprocessor have also been suggested (for reviews, see Macias et al. 2013; Pong and Gullerova 2018). The first hint of more extended roles for the Microprocessor came from the observation that DROSHA cleaves pri-miRNA-like hairpins harbored within the 5′-UTR of the mRNA encoding the DGCR8 protein itself, providing a feedback loop to control DGCR8 levels (Han et al. 2009; Kadener et al. 2009; Triboulet et al. 2009). Furthermore, the phenotypic differences observed during early T-cell development in the mouse, following *Dgcr8*/*Drosha* and *Dicer* inactivation, were correlated to transcriptomic changes that were unique to *Drosha* but not *Dicer* highlighting the existence of DROSHA-dependent, DICER-independent processing of RNAs (Chong et al. 2010). Thus, these noncanonical activities of the Microprocessor could affect cellular RNAs, beyond the described autoregulatory feedback that controls levels of DGCR8 pre-mRNA. Identification of endogenous targets for DGCR8, revealed that the Microprocessor complex binds and regulates a large variety of cellular RNAs, other than miRNAs, including mRNAs, noncoding RNAs and transcripts derived from several human active retrotransposons (LINE-1, Alu) (Macias et al. 2012; Heras et al. 2013). Accumulating evidence suggests that these noncanonical activities of the Microprocessor do indeed have physiological relevance in the turnover of cellular RNAs. For instance, DROSHA has been show to negatively regulate the expression of the transcription factor Neurogenin by cleaving evolutionarily conserved hairpins present in the *Neurogenin* mRNA that are similar to pri-miRNAs (Knuckles et al. 2012). Furthermore, miRNA-independent functions of DGCR8 were also shown to be essential for neocortical development in the mouse. This was attributed to the action of the Microprocessor directly regulating the cortical transcription factor, *Tbr1*, which also contains evolutionarily conserved hairpins that resemble miRNA precursors (Marinaro et al. 2017). Finally, DGCR8 can also associate with other nucleases, suggesting the existence of alternative DGCR8 complexes that may regulate the fate of a subset of cellular RNAs, as shown by the DGCR8-mediated cleavage of small nucleolar RNAs (snoRNAs), which functions independently of DROSHA (Macias et al. 2015).

### Cytoplasmic step of miRNA processing: DICER

In the canonical pathway, pre-miRNAs are exported to the cytoplasm and assembled into a complex containing DICER (Hutvágner et al. 2001) and the Hsp90 chaperone (Miyoshi et al. 2010). Subsequently, the pre-miRNA is cleaved by DICER, in tandem with TRBP (HIV-1 TAR RNA RBP) and PACT (protein activator of PKR) (Fig. 1A; Fukunaga et al. 2012). In this reaction, DICER serves as a molecular ruler that measures the distance from the pre-miRNA basal end to the cleavage site adjacent to the TL (also known as apical loop) (Macrae et al. 2006). This cleavage liberates the pre-miRNA TL element and creates an RNA duplex that interacts with the Argonaute 2 protein (AGO2). Of note, due to variable structural features of pre-miRNAs, the DICER-depended cleavage is often imprecise, generating two or more miRNA duplex variants that will give rise to distinct mature miRNAs (Starega-Roslan et al. 2015). The miRNA duplex is incorporated into an AGO2 protein to form the RNA-induced silencing complex (RISC), in an ATP-dependent manner with the assistance of HSC70/HSP90 chaperones (Iwasaki et al. 2010). Subsequently, AGO2 unwinds the RNA duplex and evicts the passenger strand forming the mature RISC complex (Kobayashi and Tomari 2016). The activated RISC then recognizes a specific mRNA sequence by complementary base-pairing resulting in translation inhibition and/or RNA degradation (for reviews, see Fabian et al. 2010; Iwakawa and Tomari 2015).

### Role of RBPs in the regulation of miRNA biogenesis

Due to the important role of miRNAs in the control of gene expression and organism development, the production of mature miRNAs is tightly regulated at multiple levels, including transcriptional and post-transcriptional steps. A variety of post-transcriptional mechanisms, which affect DROSHA and DICER processing, as well as miRNA modification and turnover have been previously described (Krol et al. 2010; Siomi and Siomi 2010; Finnegan and Pasquinelli 2013; Creugny et al. 2018; Treiber et al. 2018b). Dysregulation of miRNA production can result in global defects in gene expression and lead to human disease (Mendell and Olson 2012). As an example, impaired miRNA processing promotes cellular transformation and tumorigenesis (Kumar et al. 2007), and a global miRNA depletion is frequently observed in human cancers (Hata and Lieberman 2015; Lin and Gregory 2015).

Increasing evidence suggests that general RBPs, including splicing factors and other diverse RNA processing factors, act as post-transcriptional regulators of miRNA processing (for review, see Ratnadiwakara et al. 2018). In such a scenario, the binding of an RBP to the TL or a stem of a miRNA progenitor can positively or negatively affect the Microprocessor-mediated processing of pri-miRNA in the nucleus, and/or the DICER-mediated processing of a pre-miRNA in the cytoplasm (Fig. 1B,C). Below, we will discuss the role of *trans*-acting factors that bind to precursor miRNAs and influence their nuclear and cytoplasmic processing by the Microprocessor and DICER, respectively. We will also focus on the contribution of sequence variation, exemplified by single nucleotide polymorphisms (SNPs) present in the human genome that can have a role in the biogenesis of miRNAs. Finally, we will present attempts to target miRNA regulatory events using biological and synthetic compounds that could eventually lead toward the development of therapies that correct unbalanced miRNA production in disease.

### Role of LIN28 in the regulation of let-7 processing: nuclear and cytoplasmic activities

The first described example of an RBP regulating miRNA biogenesis at the post-transcriptional level involved the role of the pluripotency promoting proteins LIN28A and LIN28B in the regulation of the let-7 family of miRNAs in pluripotent embryonic stem cells (ESCs). These proteins are enriched in undifferentiated cells and their expression is gradually switched off during differentiation. LIN28 proteins harbor two RNA-binding domains, a cold shock domain (CSD) and two zinc knuckle domains that mediate recognition of the TL of let-7 in a sequence-specific manner. Binding of LIN28 proteins to let-7 precursors blocks their processing by different mechanisms at either the DROSHA and/or DICER level. Structural studies of LIN28 proteins in complex with sequences from several let-7 precursors revealed a bipartite recognition signal within the TL of let-7 precursors. The LIN28 CSD domain, which has a limited sequence specificity, binds to a let-7 closed loop to induce a conformational change of this precursor that facilitates binding of the CCHC zinc knuckles to a GGAG motif (Nam et al. 2011; Loughlin et al. 2012; Mayr et al. 2012). LIN28B binds to the TL of let-7 precursors and affects their processing by blocking the activity of the Microprocessor in the nucleus (Newman et al. 2008; Piskounova et al. 2008; Viswanathan et al. 2008). In contrast, LIN28A functions in the cytoplasm, where it recruits a TUTase (either TUT4 or TUT7) that adds a short oligo (U) stretch to the 3′-end of precursor miRNAs and blocks their processing by DICER (Heo et al. 2009). The E3 ligase TRIM25 acts as an auxiliary factor for LIN28A by binding to the let-7a precursor and stimulating TUT-mediated uridylation (Choudhury et al. 2014). Subsequently, recruitment of the 3′–5′ exoribonuclease DIS3L2 causes the degradation of the uridylated pre-let-7 (Chang et al. 2013; Ustianenko et al. 2013). This inhibitory effect of let-7 production is important to block miRNA-mediated differentiation in stem cells. LIN28A also inhibits the biogenesis of the neuro-specific miRNA-9 during neuronal differentiation of mouse cells (Nowak et al. 2014, 2017), albeit using an uridylation-independent mechanism.

### HnRNP A1 as a paradigm of an RBP regulating miRNA biogenesis in the nucleus

**FIGURE 2.**
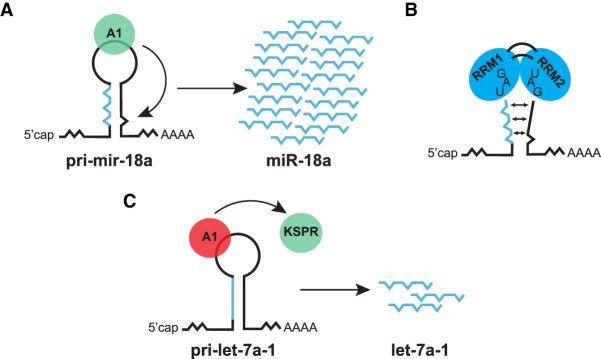
HnRNP A1 as a paradigm for RBP-mediated regulation of miRNA biogenesis in the nucleus. (*A*) Binding of hnRNP A1 to the TL of pri-mir-18a induces a structural rearrangement that results in enhanced Drosha processing. (*B*) Each RRM of hnRNP A1 recognizes an UAG motif in the TL of pri-mir-18a. (*C*) Binding of hnRNP A1 to the TL of pri-let-7 in differentiated cells outcompetes binding of the stimulatory factor, KSRP, resulting in decreased Drosha processing.

Use of an unbiased in vivo cross-linking and immunoprecipitation protocol (CLIP) searching for RNA targets of the hnRNP protein, hnRNP A1, identified the miRNA precursor, pre-mir-18a (Guil and Cáceres 2007). This miRNA is expressed as part of the miR-17-92 cluster encoded as an intronic polycistron, that includes six individual miRNAs (miR-17, 18a, 19a, 20a, 19b-1, and 92a-1) and is frequently amplified and/or overexpressed in human cancers, being also termed oncomiR-1 (He et al. 2005; Concepcion et al. 2012). Of interest, hnRNP A1 has been functionally characterized as a general RBP, with a role in many aspects of RNA processing, including alternative splicing regulation, IRES (internal ribosome entry site)-mediated translation and even telomere maintenance (Mayeda and Krainer 1992; LaBranche et al. 1998; Bonnal et al. 2005; for review, see Jean-Philippe et al. 2013). HnRNP A1 has two RNA recognition motif (RRM) domains, each harboring conserved RNP-1 and RNP-2 submotifs that represent the RNA-binding region and a C-terminal glycine-rich domain (Mayeda et al. 1994). Mechanistically, we showed that hnRNP A1 binds to the TL of pri-mir-18a and induces a relaxation at the stem–loop structure near the DROSHA cleavage site, resulting in increased Microprocessor-mediated processing (Fig. 2A,B; Michlewski et al. 2008). The processing of the other miRNAs in this cluster is not affected, indicating that hnRNP A1 acts locally to influence the processing of its target pri-miRNA. Importantly, we also observed phylogenetic sequence conservation of TL sequences of precursor miRNAs, suggesting that these sequences act as a landing pad for regulatory factors that could have a positive or negative role in miRNA production, either at the level of DROSHA and/or DICER (Michlewski et al. 2008, 2010). More recently, using an integrative structural biology approach combined with biochemical and functional assays, we were able to demonstrate that hnRNP A1 forms a 1:1 complex with pri-mir-18a, in which the tandem RRM domains of hnRNP A1 recognize two UAG motifs in the pri-mir-18a TL and the proximal stem region (Fig. 2B). This structural approach also confirmed that binding of hnRNP A1 to the TL induces an allosteric destabilization of base-pairing in the pri-mir-18a stem that promotes its processing (Kooshapur et al. 2018). Of interest, binding of hnRNP A1 to the conserved TL of a precursor miRNA does not always result in enhanced miRNA processing. We showed that hnRNP A1 binding to the TL of pri-let-7 has an inhibitory role in let-7 production in differentiated cells. This is due to a different mechanism from that shown for pri-mir-18a, and involves antagonistic roles for hnRNP A1 and another hnRNP protein, the KH-type splicing regulatory protein, KSRP, which was shown to promote let-7 biogenesis in differentiated cells (Fig. 2C; Trabucchi et al. 2009; Michlewski and Cáceres 2010). Interestingly, KSRP not only regulates the processing of let-7, but also binds to the TL of a subset of pri- and pre-miRNAs that includes miR-20, miR-26b, miR-106a, miR-21, miR-16, and promotes both DROSHA- and DICER-mediated steps (Table 1; Trabucchi et al. 2009, 2010). Thus, these findings with LIN28 and hnRNP A1 suggested a previously uncharacterized role for general RBPs as auxiliary factors that influence the processing of specific miRNAs and prompted the search for novel regulators.

**TABLE 1.**
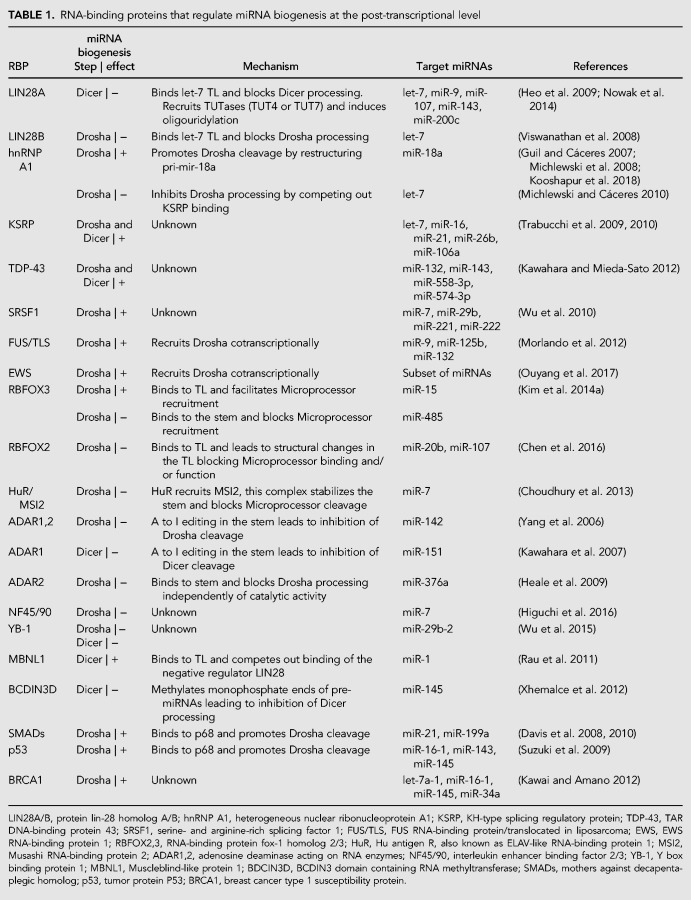
RNA-binding proteins that regulate miRNA biogenesis at the post-transcriptional level

### The terminal loop of precursor miRNAs as a hub for regulation of DROSHA and DICER activities

A flexible and long TL of approximately 10 nt was proposed to be required for efficient DROSHA processing (Zhang and Zeng 2010); however, specific sequences at the TL region of some pri- and pre-miRNAs were shown to only have a minor effect on miRNA production (Zeng and Cullen 2003; Han et al. 2006). In agreement, most precursor miRNAs display a poor phylogenetic conservation in the TL region, when compared with the high level of conservation observed in mature miRNA sequences (Berezikov et al. 2005; Akhtar et al. 2016). However, a phylogenetic analysis of human pri-miRNAs sequences revealed that approximately ∼14% (74 out of 533) of all miRNAs displayed high conservation of the TL sequence (Michlewski et al. 2008), indicating that these sequences could act as a landing platform for the binding of auxiliary factors, such as hnRNP A1, that influence the post-transcriptional regulation of miRNA production. This was further validated by the use of 2′O-methyl oligonucleotides complementary to conserved TLs, which we termed LooptomiRs (for loop-targeting oligonucleotide anti miRNAs) that block the in vitro processing of precursor miRNAs (Michlewski et al. 2008). We attributed this to a block exerted by looptomiRs on conserved sequences within the TL that are recognized by auxiliary factors required for the efficient processing of these targeted miRNAs in vitro. Conversely, it is also likely that looptomiRs could block the access of factors that negatively regulate processing of target precursor miRNAs.

In several cancers, the tumor suppressive role of let-7 is abrogated by the increased expression of its negative regulator, LIN28. Building on this concept, short, loop-targeting oligoribonucleotides were used to block binding of the negative regulator LIN28 to the precursor of let-7. These looptomiRs selectively antagonized the docking of LIN28, but still allowed the processing of pre-let-7a-2 by DICER, leading to suppression of growth in cancer cells (Roos et al. 2014). An RNA aptamer that specifically targets the pri-mir-17-92 cluster was identified through an in vitro selection process. This aptamer binds to the TL of pri-mir-18a and inhibits the biogenesis not only of miR-18a, but also of all other five miRNAs within this cluster (Lünse et al. 2010). In contrast, looptomiR-targeting miR-18a only affects the processing of this miRNA, reflecting mechanistic differences on how these two reagents influence miRNA biogenesis (Michlewski et al. 2008; Lünse et al. 2010). These evidences strongly suggest that TL recognition by RBPs could constitute a general mechanism to regulate miRNA biogenesis that operates via different mechanisms, such as altering the RNA structure of the precursor itself, recruiting additional RNA enzymes and/or affecting the recruitment and/or activity of core processing complexes associated with the Microprocessor and/or DICER. Indeed, a growing number of canonical and newly characterized RBPs have been shown to bind to TLs and regulate miRNA biogenesis (Table 1; Fig. 1B,C; Choudhury and Michlewski 2012; Castilla-Llorente et al. 2013).

### Genetic variation

**FIGURE 3.**
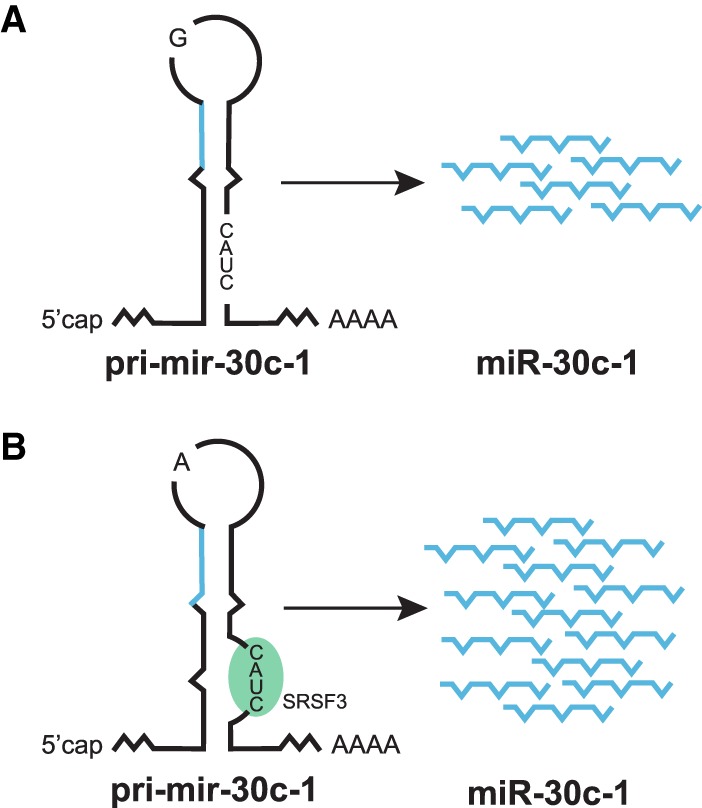
Influence of genetic variation on miRNA biogenesis. (*A*) Schematic structure of pri-mir-30c-1 with the CNNC motif (CAUC) occluded by the RNA secondary structure in the G_27_ (wild-type) variant (indicated by a G in the TL). (*B*) A single G to A substitution (A_27_) present in the TL of pri-mir-30c-1 in breast and gastric cancer patients leads to a secondary RNA structure rearrangement that facilitates binding of SRSF3 to the CAUC sequence determinant, causing increased Microprocessor-mediated processing and elevated miR-30c levels.

An increasing number of SNPs and rare mutations within precursor and/or mature miRNA sequences linked to human disease have been reported (Hogg and Harries 2014; Króliczewski et al. 2018). Despite correlations between the presence of polymorphisms in pri-and pre-miRNAs and the corresponding levels of mature miRNAs, the mechanism by which sequence variation and RNA structure control miRNA biogenesis remains mostly enigmatic. A rare genetic variation in the TL of pri-miR-30c-1 (G_27_ to A) that was found in breast and gastric cancer patients results in increased levels of mature miR-30c (Shen et al. 2009; Fernandez et al. 2017). This genetic variant directly affects the Microprocessor-mediated processing of pri-mir-30c-1 by inducing a secondary RNA structure rearrangement that opens up the pri-miRNA stem and facilitates binding of the *trans*-acting factor SRSF3 (Fernandez et al. 2017), a factor which was described to promote Microprocessor activity on a subset of miRNAs (Fig. 3; Auyeung et al. 2013). This finding raises the interesting hypothesis that primary sequence determinants in conjunction with RNA structure can act as regulators of miRNA biogenesis. Although this constitutes a largely unexplored area, the emerging picture is that human genetic variation could indeed not only affect miRNA function by targeting either miRNA-binding sites in the 3′-UTRs of target genes and/or miRNA seed sequences, but it could also have an essential role in the modulation of miRNA biogenesis (Hogg and Harries 2014). Pri-miRNA secondary structure can also influence miRNA biogenesis, as observed under limiting levels of DROSHA, when miRNAs without mismatches are processed more efficiently than mismatched miRNAs (Sperber et al. 2014). Another systematic study identified additional structural elements and sequence distribution for optimal DROSHA processing (Roden et al. 2017). This study also predicts that a small but significant fraction of human SNPs could alter pri-miRNA processing, which ultimately could influence the levels of mature miRNA and their biological function. These findings highlight the interplay between genetics, RNA structure, and post-transcriptional regulation of miRNA biogenesis.

### Auxiliary factors in post-transcriptional control of miRNA biogenesis

In addition to LIN28 proteins and hnRNP A1, several other RBPs recognize the TL of miRNA precursors and influence, either positively or negatively, their processing (Table 1; Fig. 1B,C). In the following section, we will illustrate the role of a number of RBPs in the regulation of miRNA production.

#### TPD-43

The hnRNP protein, TDP-43 (TAR DNA-binding protein-43), also promotes the processing of a subset of precursor miRNAs acting at both the level of DROSHA and DICER processing and this activity is required for neuronal outgrowth (Kawahara and Mieda-Sato 2012; Di Carlo et al. 2013). It was shown that TDP-43 is indeed a component of the Microprocessor complex (Gregory et al. 2004) and its Microprocessor-related role affects the biogenesis of a subset of at least six miRNAs, including miR-558-3p, miR-574-3p, and both strands of miR-132 and miR-143 (Kawahara and Mieda-Sato 2012). In contrast, cytoplasmic TDP-43 was shown to bind to the TL of pre-miRNAs and interact with the DICER complex (Kawahara and Mieda-Sato 2012).

#### SRSF1

The splicing factor SRSF1 (also known as SF2/ASF), which is the prototype of the SR family of splicing regulators, can also promote the maturation of a subset of miRNAs, including miR-7, miR-29b, miR-221, and miR-222. In the case of pri-mir-7, this was shown to be independent of splicing and involved SRSF1 binding to a consensus motif in the stem–loop of pri-mir-7 leading to an increase in DROSHA activity, yet the exact mechanism by which this occurs remains enigmatic (Wu et al. 2010).

#### FUS and EWS

Two members of the TET family of proteins, FUS (also known as TLS, translocated in liposarcoma) and Ewing's sarcoma (EWS), have been shown to affect miRNA biogenesis. FUS/TLS, which is associated with familial forms of amyotrophic lateral sclerosis (ALS), promotes the biogenesis of a subset of miRNAs in neuronal cells. It is recruited to chromatin, associates with DROSHA and facilitates the recruitment of the Microprocessor complex to substrate pri-miRNAs, promoting the biogenesis of neuronal miR-9, miR-125b, and miR-132 (Morlando et al. 2012). Interestingly, mutations in both TDP-43 and FUS have been linked with the etiology of ALS, suggesting a possible link between mutations in these miRNA regulators and altered miRNA biogenesis in ALS (Goodall et al. 2013; Paez-Colasante et al. 2015).

The EWS protein has a dual and opposing role in miRNA production. On the one hand, EWS has been shown to down-regulate DROSHA at the transcriptional level (Kim et al. 2014b), but it also has a positive role in miRNA production by binding to flanking sequences in the stem–loop region of pri-miRNAs and promoting the cotranscriptional recruitment of the Microprocessor to chromatin (Ouyang et al. 2017).

#### RBFOX proteins

RBFOX3 binds to a subset of pri-miRNAs and directly regulates the Microprocessor-mediated processing of selected pri-miRNAs in neuronally differentiated P19 cells and mouse brain, with stimulatory or blocking effects, depending on the miRNA (Kim et al. 2014a). Binding of RBFOX3 to the TL or to the stem of individual pri-miRNAs results in recruitment or exclusion of the Microprocessor differentially affecting the processing of the respective pri-miRNAs. Surprisingly, this role in miRNA biogenesis was independent of the cognate binding site for RBFOX3, the UGCAUG motif (Kim et al. 2014a). In contrast, a more recent study also found a role for RBFOX proteins, in the regulation of miRNA biogenesis, but in this case involved sequence-specific binding of the conserved RBFOX2 RRM to pri-mir-20b and pri-mir-107 containing the cognate motif in their TLs. This binding alters the conformation of these precursors leading to inhibition of DROSHA processing (Chen et al. 2016).

#### HuR and MSI2

Several miRNAs are expressed in a tissue- or cell type-specific manner, thereby contributing to tissue and cell identity and function (Landgraf et al. 2007). An example of an RBP determining the tissue-specific expression of a target miRNA is illustrated in the case of the brain-enriched expression of miR-7, which is processed from the ubiquitous hnRNP K pre-mRNA transcript. This brain specificity is achieved by the inhibition of pri-mir-7 processing in nonneural cells by the combined action of two RBPs, Musashi RNA-binding protein 2 (MSI2) and the Hu antigen R (HuR), which bind to the TL of pri-mir-7 (Choudhury et al. 2013). Mechanistically, HuR binds to the TL of pri-mir-7 and recruits MSI2. Both proteins act then synergistically to stabilize the pri-mir-7 stem–loop structure and inhibit Microprocessor cleavage. This is in agreement with a study showing that HuR depletion results in a significant increase of miR-7 without a noticeable change in the pri-mir-7 levels (Lebedeva et al. 2011). Notably, mature miR-7 is sequestered by a circular RNA, ciRS-7, which is primarily expressed in the cerebellum, indicating a sophisticated mechanism of miR-7 regulation (Hansen et al. 2013; Memczak et al. 2013). Other miRNAs, such as miR-505, miR-92a-1, or miR-224 are also sensitive to MSI2 and HuR depletion but the precise mechanism of action awaits further characterization (Choudhury et al. 2013). The biogenesis of miR-675 is inhibited by HuR in intestinal tissue by blocking processing of lncRNA *H19* (Zou et al. 2016). Moreover, maturation of miR-199a is blocked by HuR in hepatocellular carcinoma (HCC) in hypoxic conditions that promote glycolytic metabolism and cancer proliferation (Zhang et al. 2015). Finally, HuR inhibits processing of miR-133b from linc-MD1 noncoding RNA contributing to early stages of the muscle differentiation program (Legnini et al. 2014). It is yet to be established whether HuR controls these processes alone or in a complex with MSI2.

#### ADARs

RNA editing and RNA editing enzymes can also act to regulate miRNA biogenesis. Adenosine deaminases acting on RNA (ADARs) are responsible for the editing of adenosine residues to inosine in dsRNA. They also affect RNA interference (RNAi) and miRNA processing by deamination of specific adenosines to inosine. RNA editing of pri-mir-142, which is expressed in hematopoietic tissues, blocks its DROSHA-mediated processing. The resulting edited pri-miR-142 is subsequently degraded by Tudor-SN, a component of RISC and also a ribonuclease specific to inosine-containing dsRNAs (Yang et al. 2006). In contrast, RNA editing of pri-mir-151 blocks it processing by DICER in the cytoplasm (Kawahara et al. 2007). It was also demonstrated that ADAR proteins can influence miRNA biogenesis independently of their enzymatic activity, as evidenced by the role of ADAR2 in blocking the Drosha-mediated processing of miR-376a-2, independently of its catalytic RNA editing activity (Heale et al. 2009).

#### NF45/NF-90

The heterodimer NF45-NF90 is an RBP complex that regulates the post-transcriptional expression of a large number of cellular RNAs. It also has a negative role in the processing of pri-mir-7 in HCC. The expression of this heterodimer is elevated in primary HCC tissues compared with adjacent nontumor tissues. The NF90-NF45 heterodimer binds to pri-mir-7-1 and blocks its processing. The biological effect of this repression is the elevation of EGF receptor levels that results in the promotion of cell proliferation in HCC (Higuchi et al. 2016).

#### YB-1

The Y box-binding protein (YB-1), a member of the DNA/RNA-binding family of proteins with an evolutionarily conserved CSD, is a modulator of miRNA processing in glioblastoma multiforme (GBM). A CLIP approach revealed that YB-1 binds to the TL of pri-/pre-mir-29b-2 and regulates its processing by blocking the recruitment of the Microprocessor and DICER to its precursors. Down-regulation of miR-29b by YB-1, which is up-regulated in GBM, is crucial for cell proliferation (Wu et al. 2015).

#### MBNL1

MBNL1 stimulates the production of miR-1 by binding to a UGC motif located within the TL of pre-miR-1 and competing for the binding of the negative regulator LIN28A in the cytoplasm. In myotonic dystrophy, which is an RNA gain-of-function disease caused by expansions of CUG or CCUG repeats, MBNL1 is sequestered by these expansions. This results in a decreased miR-1 processing in heart samples from patients with myotonic dystrophy (Rau et al. 2011), contributing to pathophysiology.

#### BCDIN3D

The RNA-methyltransferase, BCDIN3D, regulates miRNA biogenesis by methylating the 5′-monophosphate end of precursor miRNAs, thus blocking the recognition of 5′-monophosphate by Dicer and inhibiting miRNA processing. In particular, it was shown that BCDIN3D phospho-dimethylates pre-mir-145 both in vitro and in vivo leading to a reduced processing by Dicer in vitro (Xhemalce et al. 2012).

### Signaling and miRNA biogenesis

Post-translational modifications of miRNA processing factors have been identified, including phosphorylation as well as ubiquitination and sumoylation that can affect DGCR8, DROSHA and/or DICER complex components (for review, see Ha and Kim 2014). It has been shown that miRNA biogenesis can also be regulated in a cell density-dependent manner (Hwang et al. 2009), and this is mediated by the tumor-suppressive Hippo pathway. At low cell density, when the Hippo signaling is suppressed, its component YAP relocalizes to the nucleus where it binds and sequesters a Microprocessor-associated component, the RNA helicase DDX17 (also known as p72), thus, down-regulating Microprocessor activity. In contrast, at high cell density the Hippo-induced cytoplasmic retention of YAP restores the association of DDX17/p72 with the Microprocessor stimulating its activity. Thus, the frequent inactivation of the Hippo pathway or expression of constitutively active YAP observed in many cancers results in a widespread miRNA suppression in cells and tumors, which explains the global down-regulation of miRNAs during cancer (Harvey et al. 2013; Mori et al. 2014).

#### SMADs

In particular, the Microprocessor-mediated step of miRNA biogenesis can be regulated by multiple signaling pathways, such as the transforming growth factor beta (TGF-β) and bone morphogenetic protein (BMP) pathways. Mechanistically, TGF-β and BMP-specific SMAD signal transducers are recruited to pri-mir-21 in a complex with the RNA helicase p68 and facilitate its DROSHA-mediated processing (Davis et al. 2008, 2010). The induction of miR-21 promotes the contractile phenotype in human vascular smooth muscle cells (VSMCs); thus, regulation of miRNA biogenesis by ligand-specific SMAD proteins acts to control the VSMC phenotype.

#### Tumor suppressors: p53 and BRCA1

As noted above, a global down-regulation of miRNAs is commonly observed in human cancers (Lin and Gregory 2015). The tumor suppressor, p53, has been shown to act as an enhancer of miRNA biogenesis in response to DNA damage, promoting the post-transcriptional maturation of a subset of miRNAs with growth-suppressive function, which includes miR-16-1, miR-143, and miR-145. Mechanistically, p53 interacts with the Microprocessor complex by binding to the DEAD-box RNA helicase p68 (also known as DDX5) in HCT116 cells and human diploid fibroblasts and promotes the processing of pri-miRNAs (Suzuki et al. 2009). The tumor suppressor breast cancer 1 protein (BRCA1) has also been shown to promote the processing of a subset of pri-miRNAs, which include let-7a-1, miR-16-1, miR-145, and miR-34a. BRCA1 interacts with components of the Microprocessor complex, namely DROSHA and the RNA helicase DDX5, as well as with SMAD3, p53, and the DHX9 RNA helicase. This novel function of BRCA1 in miRNA biogenesis could be linked to its well-established roles in tumor suppression and maintenance of genomic stability (Kawai and Amano 2012).

### Genome-wide identification of RBPs that regulate miRNA production

The initial findings that RBPs bind to TL regions of miRNAs and influence their processing, such as the examples described above (Table 1), prompted a global search for additional factors that control miRNA processing. Several strategies have been developed with the aim of identifying precursor miRNAs (both pri- and pre-miRNAs) whose biogenesis is affected by the binding of RBPs. These include the identification of additional miRNA precursors bound by cognate RBPs (Towbin et al. 2013), and identification of novel RBPs for a particular precursor miRNA sequence/s (Treiber et al. 2017, 2018a; Choudhury and Michlewski 2018). A biochemical method involving an RNA pull-down combined with SILAC mass spectrometry (RP-SMS) led to the identification of *trans*-acting factors that regulate the processing of miR-7, miR-9, and let-7 (Choudhury and Michlewski 2018). In the case of miR-9, which is specifically expressed in the brain, this approach led to the identification of LIN28A, which binds to pri-mir-9 in differentiating cells and induces the degradation of its precursor through a uridylation-independent mechanism (Nowak et al. 2014).

A recent proteomics-based pull-down approach focused on the identification of RBPs that recognize 72 different pre-miRNA hairpins used as baits in 11 different cell lines. This identified approximately 180 RBPs that interact specifically with this subset of precursor miRNAs, including known RBPs, splicing factors, as well as other mRNA processing factors (Treiber et al. 2017, 2018a). Interestingly, this approach revealed that both TLs, but also stem regions of miRNA precursors, could be specifically recognized by regulatory RBPs. In several cases, loss-of-function experiments validated the impact of these RBPs in the regulation of mRNA biogenesis, although their mechanism of action remains enigmatic. A recent study relied on a high-throughput computational screen to assess a role for 126 RBPs in miRNA biogenesis, using available eCLIP data sets from ENCODE. Those RBPs with enriched binding in the vicinity or within miR-encoding genomic loci represent candidate *trans*-acting factors for miRNA processing. This exercise resulted in the identification of 116 putative regulators that bind at 1871 annotated human precursor miRNA loci. These candidate RBPs have a potential role either positive or negative at different steps of the miRNA biogenesis cascade. Of interest, some of the interactions of individual RBPs with subsets of precursor miRNAs seem to be cell-type specific. Importantly, this difference was noted even when the corresponding pri-miRNA is expressed in both cell lines. The authors further showed that most RBPs bind fewer than 25 unique miRNA loci and in most cases, depletion of individual RBPs affects the corresponding mature miRNA levels (Nussbacher and Yeo 2018). The precise mechanisms of post-transcriptional regulation that these proteins use to control miRNA biogenesis are currently unknown.

### Role of long noncoding RNAs in the control of miRNA biogenesis

A role for lncRNAs in the post-transcriptional regulation of miRNA processing has also been recently described (Table 2). Uc.283+A, a lncRNA transcribed from an ultraconserved region, inhibits the Drosha-mediated processing of pri-mir-195. This regulatory event requires complementarity between the lower stem region of the pri-mir-195 transcript and an ultraconserved sequence in Uc.283+A. The proposed mechanism involves lower-stem strand invasion by Uc.283+A, which impairs Microprocessor recognition and blocks pri-miRNA processing (Liz et al. 2014). RNA 4 (RNCR4), a retina-specific lncRNA, stimulates the timed processing of the pri-mir-183-96-182 cluster, which is repressed at an earlier developmental stage by the RNA helicase Ddx3x, during mouse retina development (Krol et al. 2015). Finally, the heterodimer of splicing factors, NONO/PSF, which are components of the paraspeckles, bind a large number of pri-miRNAs and promote the Microprocessor-mediated processing of these precursors in HeLa cells. The lncRNA, NEAT1, interacts with NONO/PSF and scaffolds RBPs and the Microprocessor to globally promote miRNA processing (Jiang et al. 2017).

**TABLE 2.**
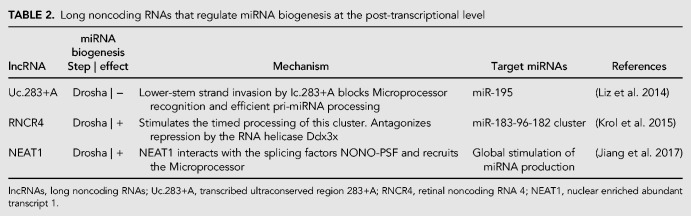
Long noncoding RNAs that regulate miRNA biogenesis at the post-transcriptional level

### Physiological relevance of miRNA regulation and human disease

Given the central role that miRNAs have in controlling the expression of target mRNAs, it is unsurprising that dysregulation of miRNA production leads to aberrant gene expression due to the misregulated expression of target mRNAs. This can affect cellular homeostasis, as well as many developmental pathways and have an impact on the development of human disease. One of the most studied examples is the altered expression of a variety of miRNAs in many different types of cancer, which arises as a consequence of mis-regulation of miRNA production, but also through the presence of mutations in miRNA processing components (for review, see Lekka and Hall 2018). Mutations in Microprocessor components, DROSHA and DGCR8 have been identified in Wilms tumors, a pediatric kidney tumor (Rakheja et al. 2014; Walz et al. 2015; Wegert et al. 2015). Conversely, DICER mutations have also been linked to several human conditions, including early childhood tumors (Heravi-Moussavi et al. 2012; Wu et al. 2013; for reviews, see Foulkes et al. 2014; Hata and Kashima 2016).

The regulation of let-7 miRNA precursors by the LIN28 proteins is a clear example of how alterations to post-transcriptional regulation of miRNA precursors can indeed lead to cancer (Viswanathan et al. 2008, 2009; Lightfoot et al. 2011). In the section below, we will discuss novel approaches used in an attempt to influence miRNA biogenesis by targeting regulatory RBPs that regulate the production of miRNAs.

### Synthetic and natural inhibitors of miRNA biogenesis

Several strategies have been developed to affect the production of miRNAs, targeting their nuclear and cytoplasmic processing machineries and/or factors that regulate their biogenesis. We have described above the use of looptomiRs and Aptamers to target the recognition of TLs by RBPs and regulate miRNA processing. Several alternative approaches have been developed to identify small molecules that bind to miRNA precursors or to RBPs that regulate miRNA biogenesis. These include the identification of a peptoid ligand that interacts with the apical loop of pri-mir-21 and inhibits cleavage by DROSHA (Diaz et al. 2014), of a benzimidazole that inhibits the biogenesis of miR-96 (Velagapudi et al. 2014) and of polyamine derivatives that block the DICER-mediated processing of pre-mir-372 processing (Staedel et al. 2018). A different strategy was developed to inhibit miRNAs that are overexpressed in human cancers, such as miR-21, in which a cyclic β-hairpin peptidomimetic binds to RNA stem–loop structures of miRNA precursors, with potent affinity and specificity. This peptide was shown to recognize the DICER cleavage site and inhibit miR-21 processing (Shortridge et al. 2017). Another study focusing on miR-21, used small molecule screening and 3D structure modeling and identified AC1MMYR2 (2,4-diamino-1, 3-diazinane-5-carbonitrile) as a potent inhibitor of pre-mir-21 cleavage by DICER (Shi et al. 2013). This inhibitor reversed epithelial-to-mesenchymal transition and suppressed tumor growth. Oleic acid (a natural monounsaturated fatty acid produced by plants and animal cells) inhibits the RNA-binding activity of Musashi RNA-binding protein 2 (MSI2), a negative regulator of pri-mir-7 processing, by binding to its N-terminal RRM (Choudhury et al. 2013; Clingman et al. 2014). Thus, the action of oleic acid could be used to disrupt the formation of a negative regulatory complex and lead to stimulation of miR-7 biogenesis (Kumar et al. 2017). Such strategies could reduce the levels of miR-7 target genes such as the EGFR oncogene, to potentially alleviate its deleterious effects in high-grade glioblastomas, where miR-7 is post-transcriptionally down-regulated (Kefas et al. 2008). Recent small-molecule screenings have identified compounds that inhibit Lin28 binding to RNA (Roos et al. 2016; Wang et al. 2018). One such compound, LI17, potently inhibited Lin28-mediated oligouridylation of pre-let-7 in vitro and in cells, causing a concomitant increase in mature let-7 levels (Wang et al. 2018). Likewise, another compound de-repressed let-7 and inhibited proliferation and stem-like properties in human cancer cells (Roos et al. 2016). These examples demonstrate that selective pharmacologic inhibition of RBPs involved in post-transcriptional regulation of miRNA biogenesis could provide a foundation for therapeutic intervention in diseases underpinned by deregulated miRNA levels.

## CONCLUSION AND PERSPECTIVES

Several layers of tightly controlled regulation have evolved to maintain the levels of mature miRNAs in order to fine-tune gene expression during development and differentiation. Such multifaceted regulation ultimately prevents gross changes in gene expression that can contribute to numerous diseases. Among these mechanisms of control, post-transcriptional steps are predominant, and increasing evidence shows the central role of general RBPs in the control of miRNA production. The binding of RBPs to TL sequences within miRNA precursors (pri- and pre-miRNAs) has emerged as a general mechanism to regulate the activity of DROSHA and/or DICER. This can encompass different mechanisms, such as conformational changes and dynamic destabilization induced by the binding of these auxiliary factors. For example, the binding of hnRNP A1 or Rbfox proteins to pri-miRNAs leads to structural changes that affect Microprocessor binding and/or activity (Table 1; Chen et al. 2016; Kooshapur et al. 2018). Another common mechanism is antagonistic binding of the regulatory RBP to either a positive or negative regulator, as seen with the competitive binding of hnRNP A1 and KSRP to let-7 precursors in differentiated cells (Michlewski and Cáceres 2010), or MBNL-1 antagonizing LIN28 binding to pri-mir-1 (Rau et al. 2011). Thus, increased knowledge of binding sites and regulatory mechanisms could facilitate the manipulation of individual miRNA expression. Similarly, recent efforts have relied on the use of oligonucleotide approaches and/or chemical or biological compounds to target the LIN28/let-7 interaction. In principle, these approaches could be expanded to compete out the binding of positive or negative regulators to individual precursor miRNAs and modify the outcome of the biogenesis pathway to correct the unbalanced miRNA levels.

Extensive genetic variation leading to altered miRNA biogenesis represents another largely unexplored mechanism of regulation. We and others have established that primary sequence determinants and RNA structure are important regulators of miRNA biogenesis. Of interest, polymorphisms and mutations within or proximal to miRNAs are frequently overlooked in disease and trait studies searching for functionally important variants, but could have an important role in determining levels of miRNA expression. In the near future, efforts will focus on the identification and functional characterization of additional RBPs or other regulators that affect miRNA biogenesis, as illustrated by recent genome-wide efforts (Treiber et al. 2017; Nussbacher and Yeo 2018). Our expanding knowledge about the mechanisms that regulate miRNA production will be essential to understand and treat human diseases that arise from deregulated gene expression.
